# Report of the Special Committee on Remedial Legislation in Regard to Expert Testimony1Presented at the thirtieth annual meeting of the Medical Association of Central New York, at Buffalo, October 19, 1897.

**Published:** 1898-02

**Authors:** Frederick Sefton

**Affiliations:** Auburn, N. Y.


### REPORT OF THE SPECIAL COMMITTEE ON REMEDIAL LEGISLATION IN REGARD TO EXPERT TESTIMONY.[1]

BY FREDERICK SEFTON, M. D., Auburn, N. Y.

YOUR special committee on remedial legislation in regard to expert testimony, appointed at the last annual meeting of this association, held at Rochester, October 20, 1896, instead of finding a dearth of material on which to frame a resolution for your action, has been favored with what might not inaptly be called a plethora of data. The amount of attention given the subject and the wealth of discussion it has provoked evidences the strength of disgust over prevailing methods, within the medical and legal professions at least, and the earnestness of intent to remedy the evils.

Out of the wealth of available material, it has been deemed expedient to embody in this report excerpts culled from some of the letters of the gentlemen of high standing in our own and the legal professions, who have kindly favored us with their view son this subject, and, in addition, to call the attention of our fellow members to the expressions of a few of the well-known writers, even at the risk of embodying quotations already familiar.

Hack Tuke, in his Dictionary of psychological medicine, says : "We find, at least as far back as the reign of Henry VIII., the courts were in the habit of summoning to their assistance, apparently as assessors, persons specially qualified to advise upon any scientific or technical question that required to be determined. Thus, on an appeal of maihem the defendant prayed the court would see the wound to see if there had been a maiming or not. And the court did not know how to adjudge, because the wound was new, and then the defendant took issue and prayed the court

1. Presented at the thirtieth annual meeting of the Medical Association of Central New York, at Buffalo, October 19, 1897.

that the maihem might be examined, on which a writ was sent to the sheriff to cause to come '*medicos chirurgicos de melioribus London, ad informandum dominum regem et curium de his quæ eis ex parte domini regis injungerentur*.'"

The English law of criminal procedure being litigious and not inquisitorial, the scientific expert was, ere long, placed in the position of an ordinary witness, subject to examination and cross-examination, and ceased entirely to exercise the function of an assessor to the legal tribunals. Our case-law contains many judicial deliverances, which it would be irrelevant to quote, and some of which do not deserve quotation, upon the untrustworthy character of medical expert evidence. The disrepute into which this class of testimony has fallen in England, appears to be due chiefly to historical causes, such as the persistency with which many medical witnesses, acting under a belief that their positions were or ought to be analogous to that of counsel, played the rôle of "medical advocates," the recklessness with which, in the opinion of lawyers, the plea of insanity and especially of moral insanity was set up in criminal cases, and, above all, the circumstances that have made the expert a witness instead of an assessor. The tendency which the legal profession displays to forget the triumphs and to remember only the failures of medical expertism, and the inheritance of witty judicial comments on the evidence of medical witnesses, which has come down to the lawyers of the present day from their professional ancestors, have done much to perpetuate a feud which is most inimical to the proper administration of justice.

Judge Doe, in State vs. Pike, 49 N. H., says: "When the authorities of the common law began to deal with insanity, they adopted the prevailing medical theories. The distinction between the duty of the court to decide questions of law and the duty of the jury to decide questions of fact, was not appreciated and observed as it now is in this state. Without any conspicuous or material partition between law and fact, without a plain demarcation between a circumscribed province of the court and an independent province of the jury, the judges gave to juries, on questions of insanity, the best opinions that the times afforded. In this manner, opinions purely medical and pathological in their character, relating entirely to questions of fact, and full of error, as medical experts now testify, passed into books of law and acquired the force of judicial decisions. Defective medical theories usurped the position of common law principles. The usurpations when detected should cease. The manifest imposture of an extinct medical theory pretending to be legal authority cannot appeal for support to our reason or even to our sympathy."

In 1874, Professor John Ordronaux said: "There is a growing tendency to look with distrust upon every form of skilled testimony. Fatal exhibitions of scientific inaccuracy and self-contradiction cannot but weaken public confidence in the value of all such evidence. If science, for a consideration, can be induced to prove anything which a litigant needs in order to sustain his side of the issue, then science is fairly open to the charge of venality and perjury, rendered the more base by the disguise of natural truth in which she robes herself."

In the *Journal of Psychological Medicine* for October, 1851, Dr. Forbes Winslow is led to express himself as follows: "Surely it is the duty of the legal profession, if they differ from the views generally propounded by medical men, to respect their opinions, however opposed they may be to those which they themselves entertain. Sad will be the consequences, if, as the results of the injudicious and uncalled-for language of the bench and the railery and misplaced wit of the bar, the public should be taught to undervalue or think lightly of the evidence of the medical psychologist in cases involving the subject of crime and insanity, responsibility and irresponsibility." That such, alas! is the unhappy tendency of events we infer from the remarks which are said to have fallen from the lips of the Lord High Chancellor of England, as referred to in *The Lancet* for August 2, 1851. We quote the following passage: "During the recent hearing of a lunacy case, the Lord Chancellor (Justice Truro) is reported to have said: 'His experience taught him there were very few cases of insanity in which any good came from the examination of medical men. Their evidence sometimes adorned a case and gave rise to very agreeable and interesting scientific discussions; but, after all, it had very little weight with the jury.'" How very "up to date" all this sounds will appeal to the experience of every member of this association who has had much to do in our courts.

In Bannister's edition of Mental medicine, E. Régis, laureate of the medico-psychological society and of the faculty of medicine of Paris, etc., quoting from the Dictionnaire encyclopedique, in effect says: "Definition of expertise (*l'expertise*).—When in a civil or criminal suit the question of dementia is raised, men of skill usually are called in, either by the judge or by the parties, sometimes to confirm, sometimes to refute the presumption or allegation of insanity. If the physician acts by virtue of a delegation of judicial authority he properly takes the title of expert; if his employment instead of being by the court is friendly and at the instance of the parties, he is a simple employé, not subject to the rules of the code of procedure. In the first case the written result of his investigations is called a report; in the second case, a consultation. Whichever way it is, at base the mission is the same, though different in origin; it tends to the same end and imposes the same duties. What applies to one, applies also to the other, in what we are about to say. First, what is an expert examination and what is an expert in the eyes of the law and in the sense of jurisprudence? Expertism is a method of instruction; its aim is to enlighten the judges in difficult, dubious or obscure cases and to furnish from special knowledge what they need in order to solve the question and make a definite judgment possible. The expert is a man of skill, charged with supplying these elements of the judgment.

"In Prussia, as well as in some other countries, the law makes it a duty of the court to call in the assistance of a medical legist to determine the mental condition of an individual. In France it is optional with the magistrates to order an examination by experts, either of his own motion or on the demand of the parties; they are the sovereign judges as to the expediency of this measure. The obligation to resort to experts is imposed upon the tribunals only in certain special matters designated by the law.

"The expertise necessarily presumes on the part of the judge, one or several definite questions addressed to the man of skill, and on his part an answer, a personal and reasoned opinion. The rôle of an expert in all its simplicity and clearly defined is: the expert is less than a judge; he is more than a mere witness: he differs from the first in that his decision has in it nothing imperative, from the second in the extent, the importance and the scientific character of his testimony. In no case should the medical expert step out of the boundaries of his proper attributes to usurp the rôle of an advocate, still less that of a judge. He should not pretend to interpret or apply the law and should be on his guard against making dangerous encroachments. Fixed animus and vain declamation fit ill in the mouth of a man who should speak exclusively in the name of science and verity. His language should be severe, cold, free from any artifice, disengaged from all interests and prepossessions. He should work for but one end, to instruct the conscience of the judges and to provide impartial decisions for the court."

Judge Foster, in the course of a most able address, delivered before the New Hampshire medical society in May, 1897, declared : "It will be observed that the most frequent and most serious complaint concerning expert testimony is the want of agreement on the same subject and in the same case, among equally learned men, rendering their testimony uncertain (it is said), confusing and bewildering, to the extent that it is unreliable and of little value ; and, yet, I doubt if an intelligent, thoughtful and candid man can be found, who will not admit that, notwithstanding all its faults and imperfections, it would be impossible to get along without it."

And, further : "It is certainly true there are and always will be differences of opinion among experts of the highest character, 'rarely in regard to well-established facts, but often in regard to probable inferences from facts ; whilst entire agreement in matters of theory and speculation would be marvelous.' But concerning this alleged misfortune, it seems hardly becoming for the legal profession to indulge in severe criticism, since there is no profession so strongly characterised by differences of opinion upon every subject,—lawyers as well as judges constantly disagreeing and the latter not unfrequently overruling one another's decisions,—unless it be the clerical profession, the members of which, it may have been observed, are not entirely unanimous in their interpretation of the holy writ. Yes, it is a visible truth that doctors, as well as lawyers and ministers of the gospel, do disagree. It would be marvelous and deplorable if they did not. If there were no disagreement, investigation and experiment would cease and science, literature and art would sink to a dead level of stupidity and laziness. If scholars and learned men had come to a condition of unanimous agreement a hundred years ago, we should have had none of the marvelous discoveries and inventions—none of the magnificent victories and triumphs in medicine and surgery—that have distinguished and illuminated the closing years of the nineteenth century."

When speaking of proposed remedial measures, in the course of his address Judge Foster refers to the efforts that have been made in Massachusetts at frequent times during the last twenty years and particularly to the latest effort in attempted legislation in that state, which was in February, 1897, when the Massachusetts medico-legal society and the Boston medico-legal society joined in the construction of a bill, which was urged before the judiciary committee of the house, but was not approved by that body. The bill provided "that the parties to any proceeding in court, before the trial, may agree upon an expert, who shall make such an examination of the case as may in his judgment be necessary and practicable, who shall give his written opinion thereon and shall attend the trial of the cause and answer such questions as may be put to him. If the parties do not agree, the court, or any judge in vacation, may appoint one or more persons learned in the branches of the science or medicine involved in the case. These persons, if more than one is appointed, shall confer and give their opinion in writing. If they do not agree they shall state in writing the points upon which they differ. If the parties do not agree, the court, upon motion of either party or upon its own motion, may appoint. The compensation of these experts shall be fixed by the court and paid by the county, but the defeated party shall refund the amount so disbursed. No medical expert shall be admitted to testify before any court, except as thus provided, and except in criminal cases, in which the defendant may call other medical expert witnesses, at his own cost, and in such case like witnesses may be called and examined by the commonwealth."

Dr. A. Walter Suiter, in a well-considered paper read at the forty-seventh annual meeting of the American medical association, cogently declared : "It is universally conceded, and justly so from the medical standpoint, that the present method of taking testimony requiring expert opinion is defective in the extreme and, in many instances, in practice absurd and ridiculous : attorneys, for example, taking advantage of the circumstances, frequently select and summon to the stand, to pose in the attitude of medical experts upon the gravest and most momentous scientific questions, members of the profession whose only special qualification consists in having agreed with their employer to express a satisfactory reply to the hypothetic statement, which supposedly covers the facts in the case at bar or under consideration. Herein lies the greatest fault in the prevailing system—namely, the absence of determining rules whereby the special qualification of the witness in a particular branch of medical science may be known and established beforehand, and the unlimited privilege which, in the usage of the courts, is possessed by attorneys and counsel to select for themselves, regardless of qualification, the witnesses whom they are to present as experts in a given case and who become *ipso*

*facto* the honest partisans of their employer, especially when he fixes their compensating fee. If reform in the presentation of medical expert testimony is ever to be accomplished, it cannot be by way of any change in the jury system of trial at present in vogue. It is much more possible that it will come through an improved and acceptable method of obtaining, and submitting for deliberation by the jury, the associated scientific facts and relations. In the judgment of the writer, the following propositions would seem to fairly comprehend the circumstances: (1) The appointment of a commission of experts by the court in each case requiring it, the experts to be especially qualified by educational experience as such. (2) The establishment of an educational curriculum and a period of service in each branch of medical science by which the qualifications of an expert witness may be known and certified. (3) Just and adequate compensation commensurate with the character of the service should be awarded and should, in every instance, in criminal cases, be paid from the public treasury upon the certificate of the presiding judge. In civil cases the compensation might or might not be fixed by the court, but should be taxed as costs to abide the event, or, by agreement, the expense might be equally divided between the contestants in the action."

Toward the close of his address, Dr. Suiter read the text of a bill which was introduced in the legislature of Minnesota in 1893, and which failed to pass. It was as follows: "*Be it enacted, etc.* Sec. 1.—In all cases pending in the courts of this state, civil or criminal, before or at time of trial of said cases, the judge of said court, when it is made to appear to him that the appointment of experts upon medical, scientific or mechanical questions is desirable, may appoint such experts to examine into the subject matter in controversy, said experts so appointed to be selected in reference to their impartiality between the contending parties; the number of such experts in each case to be fixed by the court. Sec. 2.—In all cases where experts are so appointed, the court is to fix their compensation and in all criminal cases direct the payment of the same in the same manner as witnesses on the part of the state are paid; in all civil cases the amount so fixed and determined by the court shall be taxed as disbursements by the successful party. Sec. 3.—The court may order such experts to examine into any medical, scientific or mechanical question, and after such examination to testify in court in reference thereto. Sec. 4.—The testimony of said experts so appointed by the court shall be *prima facie* evidence of the statements and conclusions as to the questions in reference to which said testimony has been given. Sec. 5.—The court may also fix and determine the amount to be allowed such experts for and on account of any medical, scientific or mechanical examination, analysis or test, which the court may deem advisable to have made, and direct the payment thereof or permit the taxation thereof as costs, as hereinbefore provided."

With the kind permission of the Hon. William Rumsey, justice of the appellate division of the supreme court, the following paragraph from a letter written by that gentleman is quoted : "With regard to a board of medical expert examiners to be appointed by the government, it does not seem to me to be advisable. In this district, in a few cases that have arisen where it has been necessary to examine the mental condition of prisoners alleged to have been insane, we have found excellent results to follow from the appointment of a commission by the court. There are throughout the state now, in the different hospitals, a good many alienists of experience and approved ability and it is so easy to get them and the results of their examinations are usually so satisfactory, that I think it would be safe to depend upon that mode of procedure for ascertaining whether or not a man should be put upon his trial. Of course, you know that in the last result a man can never be finally acquitted of a crime until he has been acquitted by a jury and the only effect of appointing a commission to examine him is to decide whether or not he is in a situation to be tried. So long as nothing else can be accomplished, it seems to me that it would be hardly worth while to go to the expense of a standing board when the necessary information can be obtained so easily by the appointment of somebody who is an alienist and competent to conclude. At the same time I think that a very considerable improvement might be made in the present procedure and that the commissioner to be appointed to examine into the sanity of a criminal should be required to be an alienist. It would be very well, I think, if the duty were devolved upon the state commission in lunacy, for instance, to designate such persons in every district as were in their judgment competent to pass upon such questions and require the selection to be made from them."

Dr. Carlos F. MacDonald, professor of mental diseases, Bellevue hospital medical college, late president of the state commission in lunacy, etc., and a physician of exceptional experience in medicolegal affairs, writes: "That the present method of obtaining medical testimony is not only imperfect, but is attended with serious evils,—evils which tend to bring the medical profession into disrepute with the public, the bar and the courts,—there can be no question. Such being the case it logically follows that there is need of reform in this matter and the question at once arises, how can that reform best be brought about? The method which, as true physicians, we would naturally adopt in a case of this kind, would be to seek out the cause of the malady and then to endeavor to secure its removal by appropriate treatment. In looking for the causes of the evil complained of we shall not have far to seek. They will readily be found, first, in the usage of courts, which permits of the selection of experts by counsel on either side, without regard to their qualifications and standing, usually the only requirement being that the expert selected is willing to give his opinion in favor of that side of the case; and, second, in the absence of any standard of qualification fixed by the medical profession as regards special study and experience in a given branch of medical science, which would, at least theoretically, render the would-be witness sufficiently skilled in that branch or subject to justly constitute him an expert.

"The first mentioned evil could doubtless be corrected by statutory provision, not for the appointment of a 'board of experts,' but for the selection of experts by the courts in each case; and, while it might occasionally happen that a judge in selecting an expert would be influenced by other than meritorious motives, I think the courts could generally be relied upon to appreciate the responsibility attaching to their action in the matter and to endeavor to select only men of skill and repute in that particular branch of medicine, men whose opinions would be respected by the bar, the jury and the general public as well. Indeed, the very fact that the experts had been thus selected would tend to give dignity to their position and weight to their opinions. The experts thus selected should be permitted to have free access to all the evidence in the case, also to examine the plaintiff or defendant, as the case may be, and then to state their opinion, whether orally or written, subject to cross-examination, the cross-examination to be limited to the matters embraced in the opinion. Their compensation, if fixed by the court, whether at a rate per diem or by fee, should be sufficient to induce qualified men to accept such service. Experts selected and paid by such a method would be free from suspicion of being biased in their opinions or influenced by pecuniary considerations, while the character and standing of those who would be likely to be selected would tend to establish confidence in their conclusions.

"Respecting the second evil, the cure for that lies in the hands of the medical profession itself—namely, to fix a standard of qualifications, based on special study and experience in a particular branch of medicine, which should entitle a member to rank as an expert in that branch, and at the same time putting its seal of disapproval and condemnation on the practice which now too frequently obtains of physicians posing as experts upon subjects respecting which they have no special knowledge or experience, relying upon their wit and the ignorance of opposing counsel and the jury to save them from exposure.

"It would doubtless simplify matters, if it were feasible, to provide that questions of expert opinion should be passed upon either by the court itself or by a jury composed of medical men, instead of, as is now the case, by a jury composed of laymen, many of whom are selected to serve in that capacity, at least in criminal cases, largely because they are lacking in knowledge and information of the matters upon which they are expected to pass. This practice, unfortunately, is one that cannot be abolished, for the reason that the terms of our federal constitution confer upon every citizen the sovereign right of trial by 'a jury of his peers.'

"Respecting the proposition to provide for the appointment of a board of examiners, either by the governor or the courts or by the commission in lunacy, it is my opinion that it would be neither desirable nor acceptable, either to the medical or legal professions or to the public, for the reason that it would savor of class legislation, which is against sound public policy. Furthermore, the prospective advantages through professional prominence and pecuniary gain, whether in fees or in salary, which an appointment on such a board would offer, would lead incompetent and unworthy members of the profession to seek appointment thereon through partisan or other questionable influences. Moreover, such a board, even if fairly representative in its make-up, would be a ready target for unfriendly or hostile criticism by their less fortunate brethren in the same line of practice, who might, whether justly or unjustly, regard themselves as being discriminated against. Then, too, it would require either a very large board or several boards to provide a sufficient number of experts in the various branches of medicine, including chemistry.

"For these reasons, it would seem to me that the most practical solution of the difficulty would be to make statutory provision for the appointment by the court of, say, from one to three experts whenever occasion arises, the law to provide that only physicians of repute in the particular branch of medical science to which the question for expert opinion relates shall be appointed, these experts to have full and free access to all the evidence in the case, as well as to the plaintiff or defendant, as the case may be, if the issue involves his mental or physical state and then to submit to the court for transmission to the jury a report in writing, setting forth their conclusion and the facts in evidence on which such conclusion is based; the cross-examination of the experts to be restricted to matters embraced within their direct statement of facts and opinion and the compensation of the experts to be fixed by the court.

"I am well aware that lawyers would raise objection to this manner of selecting experts, on the ground that a plaintiff or defendant in action is constitutionally entitled to the selection of his own witnesses and to call any witness whose testimony would tend to establish his case, a right which can neither be taken from him nor abridged short of an amendment to the constitution of the United States. To obviate this difficulty, counsel might still be permitted to call experts in addition to those selected by the court; though, it is safe to say, the opinion of the official or court-appointed experts would far outweigh any opposing opinion offered by experts selected by counsel.

"There is another point in connection with this subject which, in my judgment, should receive not only the attention of the committee, but of the society as well, for the reason that it is frequently a source of discredit to our profession. I refer to the unprofessional and, I may say, reprehensible practice of certain medical men—mostly pseudo-experts—acting as medical adviser to counsel and as expert witness in the same case. To observe, as I have often done, a medical man sitting at the elbow of counsel, actively engaged in taking notes and suggesting or preparing questions for the latter to put to the witness, also framing questions for the cross-examination of a fellow-practitioner and otherwise openly assisting the lawyer in his efforts to break down the opposing side, and subsequently taking the witness-stand himself for that purpose, is, to my mind, deplorable. If a physician is to appear as an expert witness, he should keep away from the lawyer in court and take no part in the conduct of the case which would put him in the attitude of a biased or interested party. I think a resolution should be offered to the effect that it is the sense of the society that it is derogatory to the dignity of the medical profession for any of its members to occupy the position of medical adviser to counsel in any medico-legal case in which he is to appear as an expert witness."

Dr. P. M. Wise, late superintendent of the St. Lawrence state hospital, president of the state commission in lunacy, etc., in writing of the proposed creation of a state board of medical examiners, thinks "legislation should be obtained that will replace the present unworthy, unfair and unwholesome practice of seeking and using medical expert testimony with a proper system of medical examining boards and that the organisation of a permanent state board should not be left to either the governor or the legislature. The courts should have, solely, the appointing power, as the only public officers upon whom absolute dependence could be placed as to motives actuated by partisanship or political influences. They (medical experts appointed by the courts) should receive appointments in advance of requirements, in order to eliminate beyond question any motive that may be created in a given case by the excitement of feeling of the moment; hence, permanent state expert boards are desirable. As the same difficulties exist in other branches of expert testimony, as in questions of mental responsibility, this board should have representatives of each. Moreover, this board should be empowered to call witnesses under legal restrictions, independent of counsel, cross-examine witnesses and have all facilities placed at their disposal, even to the custody of the prisoner for observation, under such conditions as they may consider necessary. The well-known Snyder case, in Washington, D. C., a few years since, made an excellent illustration of the advantages of a board thus constituted and empowered. The German system of observation of prisoners offering a plea of insanity, in a hospital for the insane, illustrates the practicability of the second feature herewith recommended. The two should be combined, when justice, as concerns the question of responsibility for crime, could balance her scales. The importance of keeping persons who commit crime, as the result of a defective organisation, under life-long restriction, is sufficiently great to be recognised by law."

Dr. George F. Shrady, editor of *The Medical Record*, etc., anent the organisation of an expert commission, believes "the most desirable plan of organisation of a commission of experts would be one that would make the commission, as such, entirely independent of the jury and give the said commission advisory power on all scientific questions. This advisory function could be exercised upon the judge, who could rule accordingly on all the purely scientific questions of fact. In order to have a reasonable representation of interests, it might be well for a commission of five to be composed as follows: one to be selected for each of the two sides and three to be named by the judge. It is obvious that no general commission could be large enough to compass all the questions involved in the difference and multiplicity of matters demanding special and expert knowledge. Therefore, it would appear more in keeping with the end to be obtained that each case should have its own commission appointed by the judge, as indicated."

Dr. Edward D. Fisher, of New York, writes: "I am in favor of some change of method in regard to expert testimony. In regard to one of the methods suggested, I am in favor of the appointment by the courts of a board of examiners. I would prefer our present method to any appointment, either by the governor or the commission in lunacy."

Dr. Samuel B. Ward, of Albany, touching on the legal difficulties to be overcome, writes: "The legal difficulties in the way appear almost insurmountable. The constitution of the state appears to guarantee to every accused individual the right to put on the stand any witness who can or will aid in clearing him and to cross-examine all witnesses. I was not aware individual rights were so sweeping when my paper (of 1889) was written. It almost seems now as if a constitutional amendment of some sort must be had before laws can become operative. Our only hope seems to lie in hammering on persistently in anticipation of eventually making some impression. Our cause is a righteous one and must finally prevail."

From the excellent article by Dr. J. B. Ransom, of Dannemora, written on a kindred subject and read before the medical society of the state of New York, February, 1895, we cull the following: "To my knowledge nothing consistent with an improved revision of the whole system of medical expert testimony has been undertaken. The question at once arises, by whom shall this needed reform be undertaken and to whom shall we look for a first movement in the right direction?

"If we listen intently, we may hear the indefinable mutterings of an arousing public sentiment in this regard and I firmly believe that the first indictment lodged by that most exacting of tribunals, an awakened public mind, will be against the medical profession and the courts will join hands with them in a mutual flagellation. The courts themselves have a way of avoiding such indictments unknown to us. Aside from this aspect of the case, there remains to us, as a profession, the much more important necessity of preserving our professional dignity and inspiring confidence in our integrity and ability. The testifying of medical experts to diametrically opposite views in a given case is not a spectacle calculated to enhance the prestige of the profession from any point of view or likely to increase the confidence of a critical public in the efficiency of our art. It then remains for us to act with promptness and determination and unite in an effort toward a change in this whole method of giving medical expert testimony.

"My own belief is that this whole system should be radically changed and the right to give medical expert testimony in any court in this state should be lodged in a nonpartisan state board, appointed by the governor and subject to approval by the senate. I will not presume upon your time, nor upon my legal acquisitions, more than to outline roughly what seems to me would be most likely to meet the requirements of the situation.

"This board should act in the capacity of instructors and advisers to the courts. It should consist of a sufficient number to provide amply for all criminal trials likely to take place at any time and should be subject to the call of the proper courts. The members of this board should be chosen from the ranks of our most expert alienists, men ripe in experience and judgment and having an ascertained qualification. The functions and powers of this board to be to examine with reference to the sanity or insanity of the accused and should, therefore, be empowered to call and examine any witnesses it may deem necessary, be they professional or lay. It should have access to all evidence throwing any light upon the case and it should also be provided that both prosecution and defense should, by showing reasonable cause, have the privilege of appealing to the courts for the calling of additional members of the said board, also to examine the accused, where the decision by the first experts called seems inconsistent. In no case should any physician, not a member of this board, be allowed to testify as an expert in insanity cases other than through the board itself and in open court his evidence should be restricted to his knowledge of the facts in the case. The board should receive its remuneration from the state. If it should be urged that the creation of a special board by the state is unwise, there seems little objection to the designation of such a board from the ranks of qualified alienists already in the state service.

"I believe it is only through some such method of procedure that the whole system of giving medical expert testimony can be purged of its incompetency, venality, sensationalism and confusion and that a trained, thoroughly experienced and efficient class of medical experts can be secured. Thereby the ends of true justice can be most nearly meted out by the courts and the medical profession even here, as in other branches of its work, sustain the high position it so deservedly holds as a conservator of the human weal."

In the course of a brief address on Medical experts and expert testimony, delivered before the association at Rochester, October 19, 1896, Dr. Sefton, of Auburn, said: "With the rise in importance of this branch of our practice, with the increase in frequency of its employment as a means of avoiding juries, or of influencing the verdicts of juries and the decisions of judges, whenever avoidance has been deemed inexpedient or become impossible, it has been demonstrated to a conviction to all thinking professional men, legal as well as medical, that the accustomed methods employed in the production of lay testimony, or testimony as to facts, is unsuited to the proper development and presentation of this special class of evidence and that a radical change is necessary.

"After some time spent in interviews and in correspondence with physicians in different localities, I have found few medical men of experience and standing who have not on more than one occasion been irritated, often have been chagrined and at times humiliated by their experience in this line of work. Complaining justly that they never are permitted to state their findings to a jury in a logical, comprehensive manner, in a manner that would enable them fully and intelligently to embody all the essential medical facts in the case to the satisfaction of mind of the average juryman, but are bound down to 'yes' and 'no' replies to categorical questions, or forced in replying to utter trite commonplaces and well-worn, stereotyped definitions, excellent sounding things, and well enough standing alone, yet completely failing to convey their real opinions, in fact, any opinions, on the case immediately at issue. Or, worse: forcing them, by limiting the scope of the answers, to appear to give an opinion opposed to what they know to be the truth, or one which, while in nowise affecting their conclusions from a medical standpoint, to a lay jury seeming to be a retraction, even a contradiction, of testimony previously adduced by them. Or when an explanatory sentence would effectually clear a cloudy point they are forbidden to make it, at least at the time; and if, further along in the case, so much of it as opposing counsel have agreed will not contravene the laws of evidence is hauled in, it comes haltingly, from a physician's standpoint emasculated of all power to make fecund with understanding the mind of the normal juryman.

"The existence of these conditions should make every one of us anxious for reform, and a reform that shall place the physician, as a witness in court, in the dignified position merited by the profession he has the honor to represent before a court of justice; that shall so restrict counsel in their dealings with experts as to make it impossible to frame an examination destined to parade a physician before court, jury and spectators as an interested, biased witness; one prone to exaggerate, eager to suppress facts against and to magnify out of all due proportions facts favoring the interests of the side in whose employ he chances to be and, necessity arising, willingly, even cheerfully perjuring himself for the sake of the remuneration promised.

"The medical expert himself: what shall be his qualifications? 'In France, in England or in Germany the courts would not recognise as an expert alienist a physician who had not had at least several years of experience in special practice and study among the insane. Here, the ease with which anybody who wishes to call himself an expert may impose upon courts and juries is astonishing. His right to be considered so is never questioned. This is the reason that experts are so readily found who will swear that a sane man is a lunatic, that a notorious lunatic is in sound mind and all his delusions facts, that a man conducting his affairs perfectly has paresis and epilepsy and that another who clearly has paresis is capable of attending to his business as usual.'

"A daily New York newspaper says editorially: 'The evils of pseudo-expertism must be indeed far-reaching when the organs of the medical profession are called upon to rebuke it. There is a prevailing lack of belief in experts nowadays among laymen, judges and juries. It is evident there must be a radical reform of some kind before the testimony of expert alienists in any given case can be accepted as always candid, honest and scientific. So long as any physician can pose as an expert if he thinks it profitable, so long as lawyers from either side may summon experts prejudiced in their favor by promise of payment for testimony in behalf of one side, just so long will expert opinions be received with incredulity and perhaps with contempt and ridicule by the public and by the juries. The medical profession owes it to itself to see that the law regarding the manner of selecting experts is changed and to repudiate all sham pseudo-experts.'

"A law might be framed compelling every physician presenting himself to the court as an expert, to prove he possesses special qualifications for the work. First, that he is a regularly graduated physician and in good standing. Second, that he has had at least five years' practical experience along the particular lines presented in the case about to be the subject of judicial investigation.

"A justice of the supreme court of the state and a number of lawyers have informed me it was the determination of many members of the bar to agree to employ but two physicians, one for each side of a case, to limit expenses and minimise chances for confusion. Such a measure is not remedial. It fails to take cognisance of, to our profession, the worst features. It fails to secure to the physician adequate opportunities for a proper physical examination of the patient. It still permits counsel to throttle the physician on the witness stand as often as the exigencies of his side of the case may seem to demand.

"It has been suggested that a law be enacted 'requiring the appointment by the judge, of a commission of one to three experts to examine and report to him upon any medical question arising in the case.' Such a change might be an improvement on existing methods, yet several objections at once suggest themselves to the mind. There would be danger that such an act would give rise to a set of court favorites composed of political doctors, so-called. If not, the difficulty of making a proper selection would be great ; 'for by what means would the judge be enabled to distinguish the true expert from the false ?'

"A law might be passed compelling opposing counsel to meet and unite in choosing a single expert as now they choose a referee. Such a law would have many advantages. Still, from the nature of the evidence demanded of an expert called upon as he is, not only for a statement of facts, but for conclusions based on those facts, he would be almost invariably at variance with one of the counsel, and be placed in as awkward and disagreeable a position as under the present methods. Besides, it might happen in important cases that it would be difficult, perhaps impossible, to secure a competent physician who would be willing to assume the responsibilities of the case alone. And there is wisdom, as well as safety to the doctor in numbers.

"Each counsel might be compelled to present the names of three competent practitioners to the court, whose duty it should be to select one name from each side, and to add a third of his own choosing, the third not to be one of the original six; the three selected to constitute a commission, to whom every possible facility should be given for making a thorough scientific examination of the patient, even permitting them to summon and examine witnesses if necessary; counsel on both sides to have free access to the commission for purposes of consultation; the commission to make a full report in writing to the court, and at the trial to testify in open court; the examination and cross-examination by counsel to be limited in its scope to the specifications contained in the written report in the hands of the presiding judge."

Believing it to be unnecessary to consume more of the time of the association, particularly in view of the fact that all procurable testimony is in accord with what precedes, and, if read, could scarcely strengthen what has been so clearly and forcibly demonstrated—namely, while your committee has met with not one dissentient voice in advocacy of a change, the expressed opinions as to what the change shall be have been many and variant. The greatest difficulty seemingly is to formulate a resolution that in no particular shall contravene or trench on the rights of the individual as guaranteed by the constitution of the state.

With no further preamble your special committee respectfully submits the following resolution for your consideration, believing, that while they are not ideal in their demands, because it would be useless to ask of the state legislature, at this time, the enactment of a law which should embrace in their entirety those reforms we all believe would fully represent the advanced state of our art, they yet contain material for much thought, and embody the nucleus of an advance on existing methods.

*Resolved,* That the Medical Association of Central New York, believing that medical expert testimony is falling rapidly into disrepute; that because of the methods pursued in the introduction of his testimony the medical expert often stands for rumor, for guesswork, for sensationalism, for partisanship, for newspaper notoriety,—instead of for study, for observation, for science, for justice and for professional tradition,—would recommend the enactment of a law by the legislature of the state, providing that hereafter no one shall be permitted to testify as a medical expert in a court of law unless possessed of a certificate issued by the regents of the University of the State of New York to the effect that he is of good character and professional standing; that he is at least thirty years of age; that he has had at least five years' special experience in the particular branches in which he desires to stand as an expert; that he has passed a regents' examination in such branches before the issuance of the certificate; that the certificate, when issued, shall be recorded in the county clerk's office where the holder resides; that medical experts shall be selected by the method at present in vogue in the courts in selecting referees; that their number shall be limited to two for each side of the case; that they shall have full access at all proper times to the person of the party to be examined and to so much of the testimony as may bear on his physical or mental condition, or both; that persons suing *in forma pauperis* shall have the benefit of a medical expert appointed by the court, placing thereby the medical profession on the same level as the legal in regard to such unfortunates; that a fee for a medical expert shall be fixed by statute and be taxable in the bill of costs, this provision, possibly, not to prevent private agreements for a compensation in addition to such stated sum; that the testimony of any medical witness called by either side to an action, not holding a certificate in accord with the above restrictions, shall be confined to evidences of fact.

In concluding, your committee wishes to record its appreciation of the courtesies extended it by the gentlemen whose views on this important subject are outlined above; to others for interesting data; and especially to Dr. J. B. Ransom, of Dannemora, for the loan of correspondence, and to J. M. Brainard, Esq., of Auburn, who as of counsel for the Lehigh Valley Railway Company has seen much of medico-legal work, for valuable suggestions.

Respectfully submitted,

FREDERICK SEFTON, Auburn, N. Y., Chairman.
ALVIN A. HUBBELL, Buffalo, N. Y.
NATHAN JACOBSON, Syracuse, N. Y.
WILLIAM S. ELY, Rochester, N. Y.
HENRY L. ELSNER, Syracuse, N. Y.

*Special Committee.*

